# *Parkin* Exon Rearrangements and Sequence Variants in *LRRK2* Mutations Carriers: Analysis on a Possible Modifier Effect on *LRRK2* Penetrance

**DOI:** 10.4061/2010/537698

**Published:** 2010-08-19

**Authors:** Paolo Solla, Antonino Cannas, Gianluca Floris, Maria Rita Murru, Daniela Corongiu, Stefania Tranquilli, Stefania Cuccu, Marcella Rolesu, Francesco Marrosu, Maria Giovanna Marrosu

**Affiliations:** ^1^Centro per i Disordini del Movimento, Dipartimento di Scienze Cardiovascolari e Neurologiche, Sezione Neurologia, Università degli studi di Cagliari, Via Ospedale 46, 09124 Cagliari, Italy; ^2^Laboratorio Centro Sclerosi Multipla, Ospedale Binaghi, Università degli studi di Cagliari, Via Is Guadazzonis, 09124 Cagliari, Italy; ^3^Dipartimento di Scienze Cardiovascolari e Neurologiche, Policlinico Universitario, Università degli studi di Cagliari, Monserrato, 09124 Cagliari, Italy

## Abstract

Mutations in *LRRK2* represent the most common causes of Parkinson's disease (PD) identified to date, but their penetrance is incomplete and probably due to the presence of other genetic or environmental factors required for development of the disease. We analyzed the presence of *parkin* sequence variants (mutations or polymorphisms) and exon rearrangements in *LRRK2* mutations carriers (both PD patients and unaffected relatives) in order to detect a possible modifier effect on penetrance. Eight families with nine PD patients with heterozygous *LRRK2* mutations (identified within 380 Sardinian PD patients screened for the presence of the five most common *LRRK2* mutations) and sixteen additional relatives were genetically investigated for the presence of *LRRK2* and *parkin* mutations. No evidence was found for the presence of pathological *parkin* mutations or exon rearrangements in patients or not affected family members. Three single-nucleotide polymorphisms (SNPs) were identified both in patients and unaffected relatives but did not significantly differ between the two groups. These data provide no support to the hypothesis whereby such *parkin* gene mutations may be commonly implicated in possible effect on penetrance in *LRRK2* mutation carriers.

## 1. Introduction

Over the last few years several disease-causing genes have been identified such as Mendelian forms of PD [1–5]. Mutations in *Leucine-rich repeat kinase 2* (*LRRK2*) gene, one of the recently identified genes, cause autosomal dominant PD [6, 7] and represent the most common causes of PD identified to date [8–11]. A complete understanding of how *LRRK2* may lead to PD, with respect to other known PD genes and putative pathogenic pathways, is still elusive [12]. Besides, penetrance of *LRRK2* mutations is incomplete [13–15] and probably due to the presence of other genetic or environmental factors required for development of the disease. Previous findings reported an interesting and unexpected interaction of *LRRK2 *with* parkin*, suggesting that a direct interaction between these two genetic causes of parkinsonism may be involved in a common pathogenic pathway [16]. In recent studies, moreover, *parkin* mutations were identified in a few patients carrying the LRRK2-G2019S mutation [17–19], suggesting a possible digenic mechanism of PD inheritance [18]. Here, we analyzed the presence of *parkin* sequence variants (point mutations or polymorphisms) and exon rearrangements in patients with Parkinson's disease (PD) having *LRRK2 *mutations and in *LRRK2* mutations carriers family members without sign of PD in order to detect possible effect on penetrance. 

## 2. Subjects and Methods

Three hundred and eighty Sardinian PD unrelated patients and two hundred and eight controls subject, consecutively recruited both from the Movement Disorders Centre of the Cagliari's University Hospital and from the Department of Neurology of the University Policlinic of Monserrato, were screened for the presence of the five most common LRRK2 mutations (G2019S, I2020T, and R1441C/G/H). Probands with evidence of *LRRK2* mutations were identified. Subsequently, these patients and their respective families were clinically studied. The probands were examined at the study site and additional family members were recruited by means of the probands. Family members who agreed to undergo genetical analysis were investigated for *LRRK2* mutations. Besides, both in patients and in family members, all exons of the *parkin* gene were analyzed for small deletions, point mutations, and rearrangements. PD patients fulfilled the diagnostic criteria for a clinical diagnosis of defined PD with at least three of the mandatory criteria (resting tremor, akinesia, and rigidity), improvement with levodopa, asymmetrical onset, and the absence of exclusion criteria. The institutional authorities at each participating site approved the study, and written informed consent was obtained from all participants.

Genomic DNA was extracted from peripheral blood samples using standard procedure. To detect the presence of the R1441C/G/H (exon31), G2019S, and I2020T (exon 41) mutations, the samples were amplified by polymerase chain reaction (PCR) using the following primers (5′ to 3′): Exon 31 Forward: TCTGAAGTCTGCTAGTTTCTC and Reverse: CTGACATTTCTAGGCAGTTG; Exon 41 Forward: GAGCACAGAATTTTTGATGCTTG and Reverse: TTTATCCCCATTCCACAGCAGTAC. PCR reactions were done in 50 *μ*l containing 1X Gold PCR buffer, 1.5 mM MgCl2, 250 *μ*M dNTPs, 25 pmoles of each primer, 2 units of AmpliTaq Gold (Applied Biosystems), and 100 ng of genomic DNA. Cycle conditions were 11 min at 94°C; 30 cycles of 30-second denaturation at 94°C; 45-second annealing at 58°C for exon 31 and 60°C for exon 41; 90-second extension at 72°C and a final extension of 7 min at 72°C. After the sizing, PCR products were denatured and blotted into Hybond-N+ membranes (Amersham Biosciences). The blots were prehybridized, hybridized with [32P]CTP labeled-probes listed below for 1 h, washed, and exposed to Kodak XAR film. All the steps were performed at the specific temperatures and time. Oligonucleotides used for the ASOs technique are indicated in Table 1. Heterozygous samples for LRRK2 mutations (R1441C or S2019G) were confirmed by PCR-RFLP using the specific endonucleases BstUI and SfcI according to the Manufacturing guides. Samples showing the loss of BstUI restriction site for R1441 were resolved by direct sequencing to identify the exact codon. The sequencing was performed using sense and antisense primers on a MEGABACE 1000 (Amersham) and results analysed with a MegaBace Sequencing Analyzer v3.0.

In all subjects coming from families with presence of *LRRK2* mutations, *parkin* mutations analysis was performed. All 12 exons of *parkin* and their intron-exon boundaries were screened for point mutations and small deletions by direct PCR sequencing. The sequencing was performed using sense and antisense primers previously described [20] on a MEGABACE 1000 (Amersham) and results analysed with a MegaBace Sequencing Analyzer v3.0.

*Parkin* exon rearrangements were also examined. Two commercially available probe kits, SALSA P051-C1 and P052-C1 Parkinson MLPA kits (MRC Holland, Amsterdam, The Netherlands; http://www.mlpa.com), were used for this assay. All exons of *parkin* were included in both kits, and the probe sequences of the same exon were different between the two kits. The assay was performed for all the 25 samples according to the manufacturer's protocol. After MLPA treatment, samples were run on an ABI 3130xl Genetic Analyzer (Applied Biosystems) with GeneScan-500 LIZ Size Standard (Applied Biosystems) according to the manufacturer's recommendations. Data were analyzed using GeneMapper software v4.0 (Applied Biosystems). Electropherograms were first visually analyzed to discard failed samples. Thereafter, peak height results were exported from Gene-Mapper to Coffalyser v9 software. Coffalyser is an Excel-based program which runs on a Microsoft Office 2003 and recommended by MRC_Holland. After normalization of data by Coffalyser macro, comparing the peak pattern obtained to that of reference samples, we obtain samples carrying aberrant copy numbers. In the Coffalyser analysis, a peak size indicates a normal copy number when showing a 0.7–1.3 ratio compared to normal controls, a deletion when showing a ratio <0.7, again when showing a ratio >1.3, and an absence when showing a ratio equal to 0. Based on this correlation, a normal peak indicates the presence of two gene copies.

Statistical analysis concerning polymorphisms of the *parkin* gene were compared between PD patients having *LRRK2* mutations and *LRRK2* mutations carriers family members without sign of PD with the Fisher's exact probability test.

## 3. Results

Eight families (six of them carrying the G2019S mutations and two carrying the R1441C mutations) with nine PD patients heterozygous carriers of *LRRK2* mutations were identified. Of these nine patients (mean PD onset 63.6 ± 10.2 years, range 44–77), seven individuals (five males and two females) carried the LRRK2 G2019S substitution and two females carried the R1441C substitution. The mean duration of disease in years for affected individuals was 8.2 ± 3.5 years (range: 1–12). Sixteen not affected proband family members (13 within families with G2019S mutation and 3 with R1441C mutation) with mean age at examination 68.6 ± 9,2 years (range, 51–83) agreed to undergo analysis for LRRK2 mutations, ten of whom (four males and six females) were found to be G2019S heterozygous carriers with no sign of PD (Table 2). No evidence was found for the presence of pathological *parkin* point mutations in anyone of the 25 subjects examined, either in PD patients or in not affected family members. Three single-nucleotide polymorphisms (SNPs) were identified: two of these SNPs reside within the intronic sequences (**IVS**2 + 25T > C = rs2075923 and **IVS**7 − 35G > A = rs3765474) while the third was the Val380Leu polymorphisms in exon 10. All these SNPs were identified both in patients and not affected family members (Table 3). Fisher's exact probability test showed that the allele and genotype frequencies of each detected *Parkin* SNPs did not significantly differ between the group of PD patients with *LRRK2* mutations and not affected family members with *LRRK2* mutations. MLPA analysis disclosed no chromosomal rearrangement for the *parkin* gene in the 25 patients confirmed in both SALSA P051-C1 and P052-C1 being ratio comprises between the cut-off values.

## 4. Discussion

To our knowledge, this is the first study which attempts to study the reduced penetrance of *LRRK2* mutations analyzing a possible digenic mechanism of PD inheritance with *parkin* mutations, studying both PD patients with *LRRK2* mutations and not affected family members with LRRK2. The detection in our families of at least ten subjects carrying the G2019S mutation in heterozygous state, with mean age at examination near to 68 years, but without any sign of PD, clearly confirmed previous observations which describe penetrance of *LRRK2* mutations as low and incomplete [13–15]. Besides, a complete understanding of how *LRRK2* gene mutation causes PD, or where it fits with respect to other known PD genes and putative pathogenic pathways, is still elusive [12]. In this scenario, both the reported interaction of LRRK2 with parkin [16] and the recent observations of several subjects simultaneously harboring both *LRRK2* and *PRKN* mutations [17–19] have invited to consider a potential genetic interplay between these two most common genetic causes of parkinsonism in the mechanisms of penetrance, with a model of gene–gene interaction. Although two previous articles have studied this combination [18, 19], they only concentrated their attention on age at onset or progression of disability, examining mainly clinical findings of PD patients, without a complete clinical and genetic familiar analysis. In fact, the first paper [18], involving 9 patients with G2019S mutations and 10 with R1441G mutations (detected among 351 patients with idiopathic PD), concluded that three detected patients harboring double mutation did not present an earlier age-at-onset or a faster progression of disease. Unfortunately, in this study a genetic analysis of the *LRRK2* mutations in relatives of these patients was not performed. In the second paper [19], regarding 138 Portuguese PD patients, 13 patients with *LRRK2* mutations (10 probands and 3 affected relatives) were identified and 2 of them carried a single heterozygous mutation in the *parkin* gene. Owing to the fact that the detected *parkin* mutation did not cosegregate in one family with PD and the PD onset age in these two siblings with and without *parkin* mutation was similar, these authors concluded that the presence of *parkin* mutations could be a coincidental finding, but a modifier effect on penetrance was not analyzed because also in this report other relatives of these patients were not studied from a genetic point of view.

Differently from these studies, therefore, the aim of our analysis was not to verify if patients with possible contemporary presence of *LRRK2* mutations and *parkin* mutations could present an earlier age-at-onset or a faster progression of disease, but, more precisely, if presence of this combination could have influenced the penetrance of PD in these patients. To this regard, our findings do not confirm the presence of *parkin* point mutations, small deletions, or exon rearrangements in our patients with *LRRK2* mutations. In fact, we did not find any pathogenic *parkin *mutations in any subjects of the 8 studied families carriers of *LRRK2* mutations. Moreover, we did not find any associations between three different polymorphisms of the *parkin* gene and the reduced penetrance of *LRRK2* mutations in these families, indicating that *parkin* mutations are not commonly involved in determining the pathogenesis of PD in these patients. Our data, together with the two previous studies [18, 19], exclude the occurrence of pathogenic *parkin* mutations in the majority of PD patients with LRRK2 substitutions.

In conclusion, our findings do not provide support to the hypothesis whereby *parkin* gene mutations may be commonly implicated in possible modifier effect on penetrance in *LRRK2* mutation carriers.

## Figures and Tables

**Table 1:** Oligonucleotides used for the ASOs technique. The nucleotide change in the oligonucleotides is underlined.

Exon 31	1441 wild-type allele R	5′*-CCCTCCAGGCTCGCGCTTC-*3′
	1441 mutant allele C	5′*-CCCTCCAGGCTTGCGCTTC-*3′
	1441 mutant allele G	5′*-CCCTCCAGGCTGGCGCTTC-*3′
	1441 mutant allele H	5′*-CCCTCCAGGCTCACGCTTC-*3′

Exon 41	2019 wild-type allele G	5′*-AAAGATTGCTGACTACGGCA-*3
	2019 mutant allele S	5′*-AAAGATTGCTGACTACAGCA-*3′
	2020 wild-type allele I	5′*-GCATTGCTCAGTACTGCTGT-*3′
	2020 mutant allele T	5′*-GCACTGCTCAGTACTGCTGT-*3′

**Table 2:** Study of families with LRRK2 mutations.

Subjects with	PD Patients with LRRK2 mutations	Not affected relatives with LRRK2 mutations	Not affected relatives without LRRK2 mutations
G2019S	**7**	**10**	**3**
Mutation	(5 M/2 F)	(4 M/6 F)	(1 M/2 F)
R1441C	**2**	**0**	**3**
Mutation	(2 F)		(2 M/1 F)
Total	**9**	**10**	**6**
(25)	(5 M/4 F)	(4 M/6 F)	(3 M/3 F)

M: Male; F: Female.

**Table 3:** Allele and genotype frequency distribution of three single-nucleotide polymorphisms of the parkin gene in carriers of LRRK2 mutations (R1441C or G2019S) with or without PD.

Polymorphism	R1441C Affected	G2019S Affected	Total R1441 or G2019 Affected	G2019S Healthy	*P *=
rs2075923 IVS2+25T→C					
Allele T	2	9	11	16	.243
Allele C	2	5	7	4	
Genotype TT [Mean AO or AE ± SD (ys)]	0	3 [65.5 ± 9.0]	3 [65.5 ± 9.0]	7 [67.7 ± 9.9]	
Genotype TC [Mean AO or AE ± SD (ys)]	2 [67.8 ± 7.5]	3 [78.7 ± 4.2]	5 [74.4 ± 6.6]	2 [68.2 ± 8.2]	
Genotype CC [Mean AO or AE ± SD (ys)]	0 [NV]	1 [77.2]	1 [77.2]	1 [69.7]	
rs3765474 IVS7−35G→A					
Allele G	2	8	10	11	.550
Allele A	2	6	8	9	
Genotype GG [Mean AO or AE ± SD (ys)]	1 [60]	2 [73.9 ± 1.4]	3 [65.5 ± 4.9]	2 [73.4 ± 0.7]	
Genotype GA [Mean AO or AE ± SD (ys)]	0	4 [64.3 ± 15.9]	4 [64.3 ± 15.9]	7 [67.2 ± 9.7]	
Genotype AA [Mean AO or AE ± SD (ys)]	1 [65]	1 [59]	2 [62 ± 4.2]	1 [62.5]	
EX10 c.1239G>C Val380Leu					
Allele G	3	11	14	14	.656
Allele C	1	3	4	6	
Genotype GG [Mean AO or AE ± SD (ys)]	1 [60]	4 [61.3 ± 13.7]	5 [61.0 ± 11.9]	4 [60.9 ± 7.7]	
Genotype GC [Mean AO or AE ± SD (ys)]	1 [59]	3 [77 ± 5.4]	4 [74.8 ± 6.2]	6 [72.7 ± 5.5]	
Genotype CC [Mean AO or AE ± SD (ys)]	0	0	0	0	

AO: age at disease onset; AE: age at examination; SD: Standard deviation; ys: years.
